# mTOR Signaling in Metabolic Stress Adaptation

**DOI:** 10.3390/biom11050681

**Published:** 2021-05-01

**Authors:** Cheng-Wei Wu, Kenneth B. Storey

**Affiliations:** 1 Department of Veterinary Biomedical Sciences, Western College of Veterinary Medicine, 52 Campus Drive, University of Saskatchewan, Saskatoon, SK S7N 5B4, Canada; 2 Toxicology Centre, University of Saskatchewan, Saskatoon, SK S7N 5B3, Canada; 3 Institute of Biochemistry and Department of Biology, Carleton University, 1125 Colonel By Drive, Ottawa, ON K1S 5B6, Canada; KennethStorey@cunet.carleton.ca

**Keywords:** cell signaling, protein translation, environmental stress, TOR, metabolism, Akt, hibernation, estivation, anoxia, hypoxia, dauer

## Abstract

The mechanistic target of rapamycin (mTOR) is a central regulator of cellular homeostasis that integrates environmental and nutrient signals to control cell growth and survival. Over the past two decades, extensive studies of mTOR have implicated the importance of this protein complex in regulating a broad range of metabolic functions, as well as its role in the progression of various human diseases. Recently, mTOR has emerged as a key signaling molecule in regulating animal entry into a hypometabolic state as a survival strategy in response to environmental stress. Here, we review current knowledge of the role that mTOR plays in contributing to natural hypometabolic states such as hibernation, estivation, hypoxia/anoxia tolerance, and dauer diapause. Studies across a diverse range of animal species reveal that mTOR exhibits unique regulatory patterns in an environmental stressor-dependent manner. We discuss how key signaling proteins within the mTOR signaling pathways are regulated in different animal models of stress, and describe how each of these regulations uniquely contribute to promoting animal survival in a hypometabolic state.

## 1. Historical Background

Rapamycin, a macrolide compound isolated in the 1970s from Easter Island, was originally characterized for its efficacy as an antifungal against *Candida albicans* [1]. Beyond its antifungal properties, rapamycin became a compound of interest as it was also shown to be a potent immune and tumor suppressant [2,3]. However, it was not until the 1990s when rapamycin’s mechanism of action began to be elucidated. First, it was determined that rapamycin inhibited the activity of an immunosuppressive compound called FK506 in part by competing for its binding towards FKBP (FK506 binding protein), and this interaction facilitated the inhibition of T cell activation [4]. It became apparent that interactions with FKBP are essential for rapamycin activity, and the rapamycin-FKBP complex was shown to reduce cell growth and DNA synthesis in part by inhibiting the growth-regulating 70 kd S6 protein kinase (P70S6K) [5]. In subsequent affinity purification studies using the rapamycin-FKBP complex, a serine/threonine protein kinase initially termed RAFT1 (rapamycin and FKBP12 targets 1) or FRAP (FKBP-rapamycin-associated protein) was identified as the principal protein responsible for rapamycin’s biological effects. This RAFT1/FRAP was homologous to the yeast TOR (target of rapamycin) protein identified previously in a rapamycin resistant genetic screen [6]. RAFT1/FRAP is now officially referred to as the mechanistic target of rapamycin, mTOR, renamed recently from the mammalian target of rapamycin, perhaps due to its evolutionarily conserved function in yeast, flies, worms, and mammals [7,8,9]. 

Functionally, mTOR exists in a protein complex and acts on different downstream targets depending on its interactions with different regulatory proteins. Two functionally distinct mTOR complexes, mTORC1 and mTORC2, are formed based on mTOR’s interaction with two mutually exclusive binding partners that influence its substrate recruitment and subcellular localization [10]. These two proteins are Raptor (regulatory associated protein of mTOR) that form mTORC1 and Rictor (Rapamycin-insensitive companion of mammalian target of rapamycin) that form mTORC2. As implied in Rictor’s name, the mTORC2 complex is insensitive to the effects of rapamycin as it was determined that the rapamycin-FKBP complex only binds to mTORC1 but not mTORC2 [11]. Both mTORC1 and mTORC2 exert their biological effects by phosphorylating various downstream targets. In general, mTORC1 contributes to cell growth and metabolic regulation whereas mTORC2 has well-characterized functions in the regulation of cytoskeletal remodeling and cell survival [12,13]. 

A central feature of mTORC1 is its ability to sense and relay changes in environmental cues (i.e., nutrient depletion, stress, growth factors) into downstream signals that affect cell growth and metabolic functions [12]. The wide-ranging regulatory roles of mTOR in metabolic homeostasis have established this protein complex as a central controller of various physiological functions, and dysregulation of mTOR has been linked to disease states including cancer [12]. Beyond human health research, studies of the mechanisms of mTORC1 regulation have also explored the roles of mTORC1 in mediating animal adaptation to various environmental stresses in nature. Due to mTORC1’s unique ability to exert metabolic changes based on external cellular cues, dynamic control of mTORC1 activity has recently emerged as an important regulator contributing to the establishment of a natural hypometabolic state in animals under environmental stress. Metabolic depression is an adaptive survival strategy utilized by many animal species when faced with unfavorable or temporary uninhabitable environments [14]. Examples of these include, but are not limited to, exposure to prolonged high or low temperatures, limited food or water availability, and oxygen deprivation. To survive such environmental challenges, select animal species can depress their metabolic rate, often to just 5–40% of their resting rate, and enter a state of dormancy aimed at conserving cellular energy reserves in order to promote survival. In this review, we will focus specifically on the functions of mTORC1 concerning its role in regulating metabolic adaptation to natural environmental stresses. In the past decade, we and others have identified mTORC1 as a key regulator governing animal survival under various environment-induced situations of metabolic rate depression. These have included hibernation in mammals [15,16], anoxia-tolerance of submerged turtles [17], hypoxia-tolerance of naked mole rats [18], and dehydration-induced estivation in frogs [19]. A surprising finding across these studies is that mTORC1 can be both suppressed and activated during periods of metabolic depression, suggesting that its functional role is stress-type and species-dependent. In this review, we will discuss examples of how differential mTORC1 regulation contributes to animal survival across different types of environmental stressors. 

## 2. mTORC1 Control of Cell Growth under Metabolic Stress

**Figure 1:**
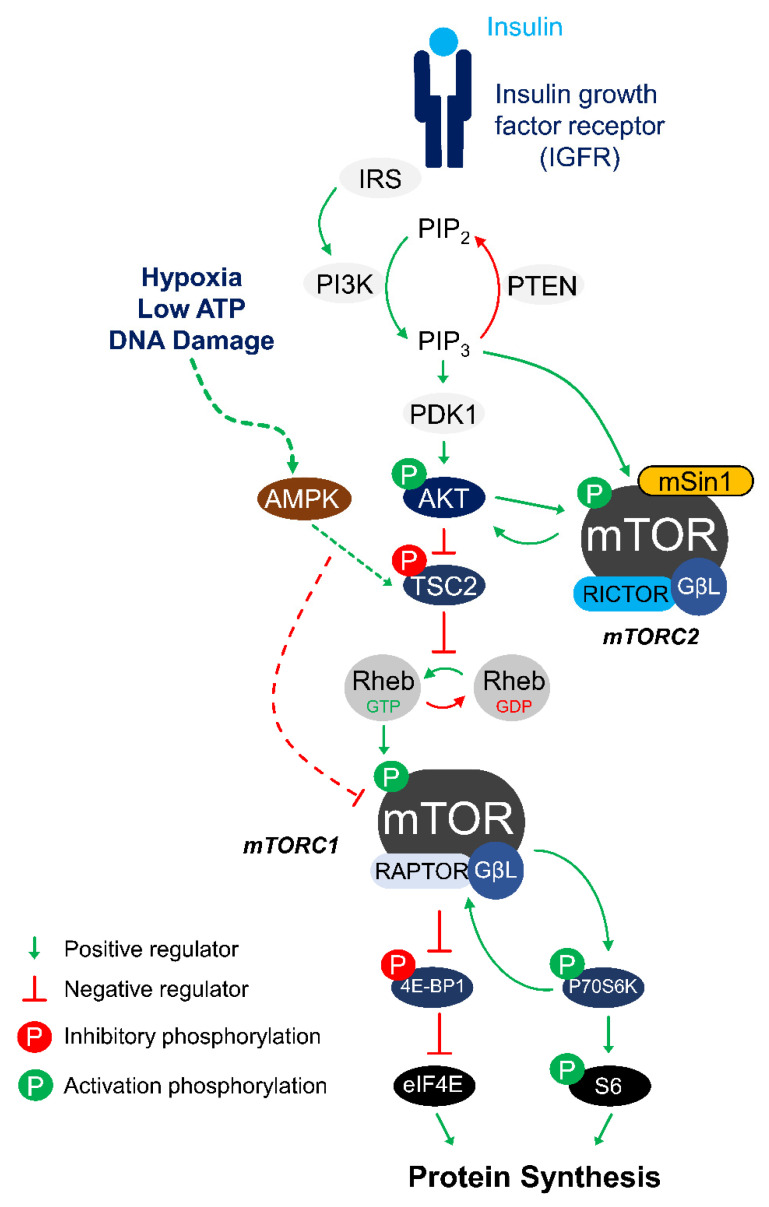
The mTOR signaling pathway. In response to insulin stimuli, IGFR activates the IRS that leads to the phosphorylation and activation of Akt. Activated Akt phosphorylates TSC2 to suppress its inhibitory effect on Rheb-GTP recycling from Rheb-GDP that is required for mTOR activation. Rheb-GTP can then activate the mTORC1 complex (mTOR, Raptor and GβL) to exert control over protein synthesis by phosphorylating 4E-BP and P70S6K, which are two key regulators of ribosome assembly. Under periods of cellular stress such as hypoxia, low ATP, or DNA damage, AMP kinase acts to inhibit mTORC1 by activating the TSC2 protein in human cells. The mTORC2 complex can be activated at its regulator subunit mSin1 by Akt or by PI3K-generated PIP3. Activated mTORC2 phosphorylates Akt in a positive feedback loop. Abbreviations are: IGFR, Insulin growth factor receptor; IRS, Insulin receptor substrate; PI3K, Phosphoinositide 3-kinase; PTEN, Phosphatase and tensin homolog; PIP2, Phosphatidylinositol (4,5)-bisphosphate; PIP3, Phosphatidylinositol (3,4,5)-trisphosphate; TSC2, Tuberous sclerosis complex 2; Rheb, Ras homolog enriched in brain; mTOR, Mechanistic target of rapamycin; Raptor, Regulatory-associated protein of mTOR; mSin1, mammalian SAPK interacting protein 1; Rictor, rapamycin insensitive companion of mTOR; GβL, G protein beta subunit-like; 4E-BP, Eukaryotic translation initiation factor 4E-binding protein 1; eIF4E, Eukaryotic translation initiation factor 4E; P70S6K, Ribosomal protein S6 kinase beta-1; S6, Ribosomal protein S6.

The molecular pathway by which mTORC1 converts upstream signals such as growth factors and amino acid availability into downstream responses is well established and briefly illustrated in Figure 1. In animals, mTORC1 upstream signals are largely governed by dietary intake of nutrients including glucose and amino acids; in general, mTORC1 activity is stimulated by feeding and inhibited by fasting [12]. In addition to nutrient cues, mTORC1 is also negatively regulated by the AMP-activated protein kinase (AMPK) under stressful cellular environments including low ATP levels, low oxygen levels (hypoxia), or the presence of DNA damage [20,21,22]. One of the first downstream targets of mTORC1 identified was P70S6K; treatment with rapamycin blocked the phosphorylation and activation of P70S6K, leading to cell cycle arrest [5]. Activated P70S6K phosphorylates various substrates including the 40S ribosomal S6 protein and the eukaryotic translation initiation factor 4B (eIF4B), both of which are required for assembling an active protein translation machinery [23]. Next, mTORC1 was also shown to regulate protein translation via phosphorylation and inactivation of the eukaryotic initiation factor 4E binding protein (4E-BP1). When phosphorylated, 4E-BP1 dissociates from eIF4E, permitting an interaction between eIF4E and eIF4G to form part of the 5’ cap-binding complex [13,24]. Through its control over P70S6K and 4E-BP1, mTORC1 directly regulates protein synthesis, which is one of the most ATP-expensive cellular processes and is highly sensitive to energy supply [25]. 

### 2.1. mTORC1 Regulation in Hibernation

**Figure 2:**
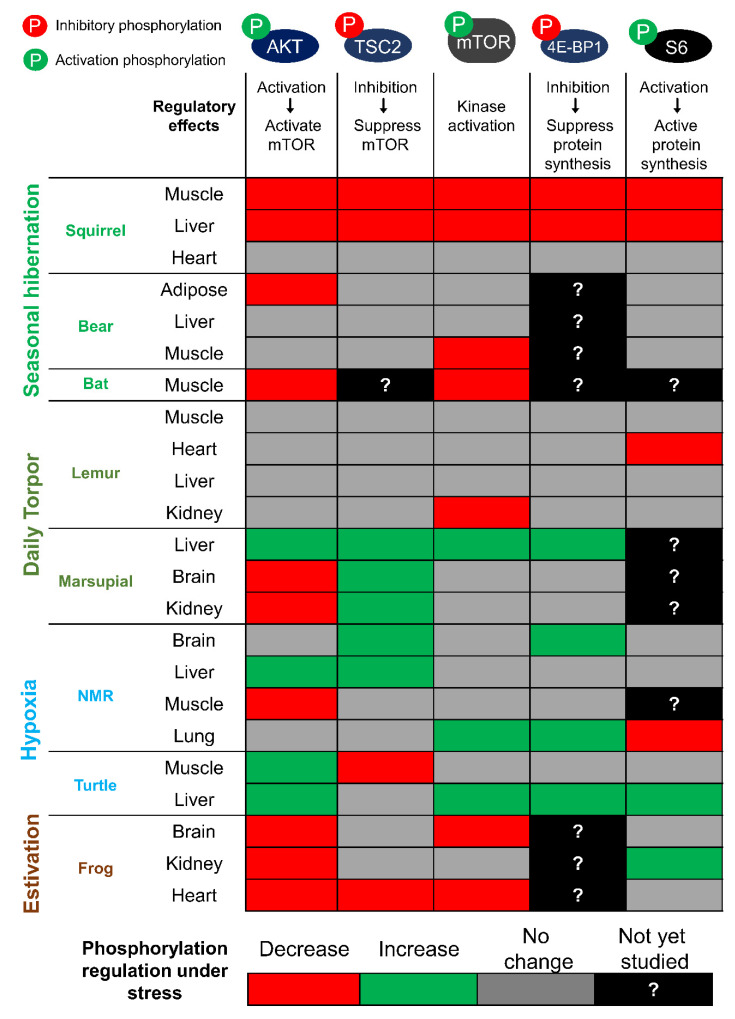
Regulatory pattern of mTORC1 signaling proteins across different animal models of hypometabolic stress. Summary of p-Akt, p-TSC2, p-mTOR, p-4E-BP1, and p-S6 regulation across different animal species under metabolic stress conditions discussed in this review. Phosphorylation (P) in green indicates the activation effect while phosphorylation in red indicates inhibitory effect. Data are summarized from the following studies: [15,16,17,18,19,46,51,52].

**Figure 3:**
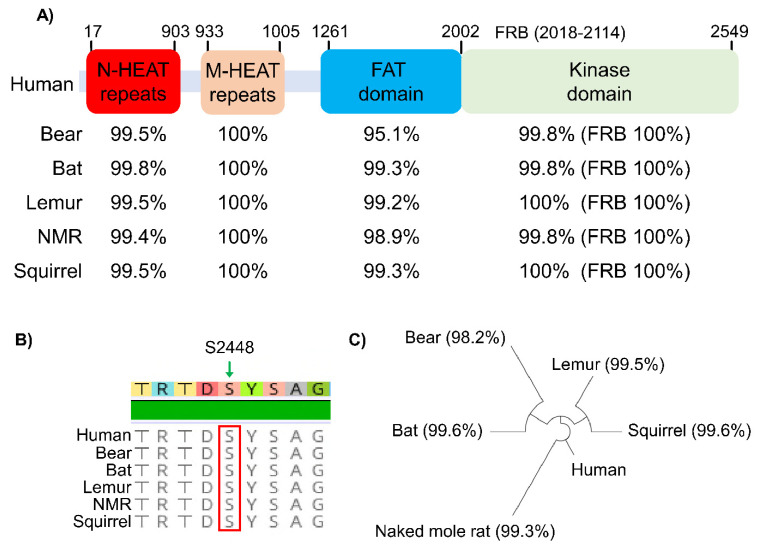
mTOR is highly conserved across mammals. (**A**) Diagram depicting domains of the human mTOR protein and relative conservation of each domain across different mammalian species. NMR: naked mole rat; N-HEAT: N-terminal Huntingtin, EF3, PP2A, and TOR1; M-HEAT: middle-HEAT; FAT: FRAP, ATM, TRRAP; FRB: FKBP12–Rapamycin Binding. Figure is adapted from Yang et al. 2017 [26]. (**B**) The key mTOR phosphorylation residue Ser-2448 and it flanking amino acids is 100% conserved across different mammalian species. (**C**) Lineage tree of mTOR protein between human, squirrel, lemur, bear, bat, and naked mole rat.

The interest in studying mTORC1 in hypometabolic animal models largely centered on the well-characterized role of mTORC1 in regulating protein biosynthesis. In Figure 2, we present a summary of key proteins of the mTORC1 signaling pathway and how they are regulated in different tissues across multiple animal species that endure environmental stress conditions. Studies of mTOR across diverse animal species are possible since the core mTOR protein, its identified domains, and key phosphorylation sites are all highly conserved compared to the human homologue, as illustrated in Figure 3 for six mammalian species. The N-terminal HEAT (Huntingtin, EF3, PP2A, and TOR1) repeat domain of mTOR facilitates interaction with either the Raptor or Rictor protein in forming the mTORC1 or mTORC2 complex. Activity of mTOR is negatively regulated at the FAT (FRAP, ATM and TRRAP) domain by a protein named DEPTOR (DEP-domain-containing mTOR-interacting protein) and is positively regulated at its kinase domain by GβL (G protein β-subunit like protein). Within the kinase domain lies the FRB (FKBP12-rapamycin binding) region that facilitates the inhibitory binding of FKBP12-rapamycin to mTORC1 [26]. Given that all of the identified domains are 95–100% conserved in six different mammals (Figure 3A), it is expected that the mechanism of mTOR regulation in these animals would be also be highly conserved.

The 13-lined ground squirrel (*Ictidomys tridecemlineatus*) is a well-studied model animal that undergoes obligatory hibernation in the winter. The hibernation season involves multiple bouts of torpor where the animals undergo reversible global arrest of metabolic and physiological functions that include a reduction in core body temperature (from 37 °C to ~5 °C), suppression of respiration (>40 to <1 breath/min), and reduction in heart rate (from 350–400 to 5–10 bpm) [27,28]. An initial study using quantitative autoradiographic ^14^C leucine incorporation demonstrated that protein translation was severely reduced in the brain of ground squirrels that are in torpor, and this is supported by a decrease in active ribosome content as determined by polysome profiling [29]. As mentioned earlier, protein translation is one of the most ATP-consuming processes in the cell, and its suppression during torpor is hypothesized to be a crucial factor in establishing a hypometabolic state. Whereas the drop in body temperature undoubtedly contributes to the suppression of protein synthesis at a low body temperature, a direct increase in phosphorylated eIF2α (Ser-51) content during torpor shows that inhibition of protein synthesis is also actively regulated [29]. Building on these findings, subsequent studies began to characterize the regulatory patterns of other key proteins involved in protein synthesis to understand the biochemical basis of translational control during torpor. 

The Akt protein is a positive regulator of mTORC1 that is activated by the insulin/insulin-like growth factor 1 receptor (IGFR) in response to insulin stimulation (Figure 1) [30]. This protein kinase phosphorylates a large number of downstream targets to regulate different cell functions including cell survival, proliferation, growth, and metabolism [30]. With respect to mTORC1, Akt acts on the tuberous sclerosis complex 2 (TSC2) protein that serves as a principal suppressor of mTORC1 [31]. As a molecular inhibitor for mTORC1, TSC2 is a GTPase-activating protein that converts the GTPase Rheb into its inactive GDP-bound form that prevents Rheb-dependent mTORC1 activation. When TSC2 is phosphorylated by Akt (Thr-1462) in response to IGF-1 activation, it dissociates from Rheb to release its inhibition and thereby permits Rheb activation of mTORC1 via an allosteric mechanism involving the realignment of mTOR active site residues [26,32,33]. How Akt is regulated during ground squirrel hibernation has been the subject of several studies. The consensus finding is that Akt activity is highly suppressed during torpor. The evidence supporting this includes a significant reduction in the phosphorylation state of the Akt activating residue Ser-473 [16,34,35,36,37]. Enzymatic assays have also shown that Akt purified from tissue of hibernating ground squirrels is significantly less active as compared to the enzyme from euthermic control ground squirrels [34,38]. Consistent with reduced Akt activity, the TSC2 phosphorylation (Thr-1462) level is significantly decreased by ~50% in the skeletal muscle of ground squirrels while in torpor [16]. When activated, mTOR can be phosphorylated at several residues including Thr-2446, Ser-2448, and Ser-2481 [39]. Initially, phosphorylation at Ser-2448 was thought to be catalyzed by Akt due to the requirement of PI3K for this process; however, it was later shown that phosphorylation at Ser-2448 is catalyzed by the mTORC1 downstream target P70S6K [39,40]. Levels of p-mTOR (Ser-2448) have been shown to be significantly reduced across multiple ground squirrel tissues during torpor including the skeletal muscle, liver, and kidney [16,37,41]. Beyond TSC2, mTORC1 is positively regulated by a protein named GβL that stabilizes the mTOR kinase domain, and GβL is required for the mTOR-Raptor complex to respond to nutrient cues [42]. During torpor, protein expression of GβL was down-regulated by 31% in skeletal muscle of ground squirrels [16]. Consistent with mTORC1 being inactive during torpor, phosphorylation of mTORC1 downstream targets 4E-BP1 (Thr-36) and S6 protein (Ser-235) were also significantly reduced [16]. Collectively, these findings support a model where mTORC1 activity is actively suppressed in hibernation via a combination of post-translational (p-mTOR, p-TSC2) and translational (GβL) mechanisms that serve to inhibit protein synthesis by negatively regulating the assembly of protein translation machinery (4E-BP1, S6 protein). 

As ground squirrels emerge from torpor, the initial arousal phase is characterized by a rapid rise in body temperature along with an increase in respiration and heart rate. In this metabolically re-engaged phase, the pattern of mTORC1 suppression observed during torpor is completely reversed in ground squirrel skeletal muscle. During the early arousal phase, p-mTOR (Ser-2448), p-TSC2 (Thr-1462), and total expression of GβL are all highly elevated and are at an even higher level than measured in active euthermic animals [16]. This period of hyper-mTORC1 activation appears to be brief as the activity of mTORC1 components falls back to euthermic levels during the interbout arousal stage of ~1–3 days before animals descend again into torpor. Intriguingly, while this pattern of reversible mTORC1 depression and re-activation is generally observed in other ground squirrel tissues such as the liver and kidney, cardiac muscle is an exception [41]. In the heart, p-mTOR, p-TSC2, and GβL are not suppressed during torpor as compared to euthermia. Consistently, there were no changes in levels of p-4E-BP1 or p-S6 in cardiac muscle during torpor [16], suggesting that mTORC1 is functioning at normal levels during torpor despite the drastic drop in metabolic rate. This muscle type-specific regulation of mTORC1 may be explained in part by the fact that heart beat must continue during torpor and, indeed, cardiac muscle shows hypertrophy (~21% increase in tissue mass) that increases cardiac power output to compensate for a decreased heart rate and increased power needed to circulate cold blood [43,44,45]. While ground squirrels remain the best-characterized model of mammalian hibernation, mTOR has also been analyzed in other mammalian species undergoing torpor. For example, in the hibernating greater tube-nosed bat *Murina leucogaster*, p-mTOR levels are reduced by ~50% during torpor. This is characterized by a biphasic oscillation of p-mTOR levels that is similar to the pattern observed in ground squirrels where p-mTOR in bats is at its lowest at about 7 days into a hibernation bout but is followed by a sudden increase when animals enter the arousal phase [15]. Consistent with Akt acting upstream of mTOR, p-Akt levels were also significantly reduced in *M. leucogaster* during torpor. For both mTOR and Akt, the total protein levels did not fluctuate in bats during hibernation (as also seen in ground squirrels), suggesting that post-translational phosphorylation was the primary mechanism of regulation [15]. Seasonal regulation of p-mTOR is also observed in the hibernating grizzly bears, *Ursus arctos horribilis*. Levels of p-mTOR in grizzly bear muscle biopsies were significantly higher in the months of October when the animals undergo hyperphagia to increase body mass than in January when the animals were dormant in hibernation [46]. A study in the Japanese black bear *Ursus thibetanus japonicas* showed that phosphorylation levels of the mTORC1 downstream target P70S6K was significantly increased in April during post-hibernation in comparison to November (pre-hibernation), suggesting that mTORC1 signal is enhanced following hibernation [47]. Collectively, these studies indicated that mTORC1 is generally suppressed in animals during seasonal hibernation and re-activated/enhanced post-hibernation. This oscillation pattern of mTOR regulation may function to support the establishment of a hypometabolic state in order to promote survival in seasonal hibernators that can go for months without food and water. 

Interestingly, recent studies have provided new evidence to suggest that alternative patterns of mTORC1 regulation may exist in non-seasonal/daily hibernators (Figure 2). The grey mouse lemur, *Microcebus murinus*, and the South American marsupial, *Dromiciops gliroides*, are examples of tropical/sub-tropical mammals that undergo daily or multi-daily torpor bouts in response to transient food shortage or low ambient temperature [48,49,50]. A recent study that examined the regulation of the insulin signaling pathway across six different lemur tissues during torpor showed that were no changes to the levels of p-Akt (Ser-473) and only one out of six tissues (kidney) displayed a slight reduction in p-mTOR (Ser-2448) levels [51]. Similar trends were also observed in marsupials during daily torpor where no reduction in p-mTOR (Ser-2448) was evident but, surprisingly, levels of p-mTOR in liver were actually slightly elevated compared with the torpid state [52]. One possible explanation for such discrepancy in mTORC1 regulation amongst hibernators may be associated with the type of torpor. Lemurs and marsupials primarily undergo shallow daily or multi-daily torpor bouts while maintaining relatively high body temperatures ranging from ~15–18 °C [49,53]. This contrasts with animals such as ground squirrels or bats that often register core body temperatures close to 0 °C [54,55]. It is also possible that the reduction of mTORC1 in seasonal hibernators such as bats and squirrels is related to the duration of the torpor bouts, since these animals can remain in a hypometabolic torpid state for several weeks to a month at a time. This is in contrast to torpor strategies utilized by lemurs and marsupials that often last only ~12 hours or a few days at a time. The upkeep of mTORC1 activity during daily torpor may be an important mechanism in maintaining a relatively high body temperature in these hibernators. This is supported by studies in mice that demonstrated that mTORC1 in liver is an important contributor to body temperature regulation, presumably via its positive roles in promoting various metabolic processes [56]. Consistent with the idea that mTORC1 activity is preserved during daily torpor, a recent study revealed that 23 different microRNAs (miRNA) that are predicted to inhibit various components of the mTORC1 signaling pathway are significantly reduced in the liver of marsupials during torpor [57]. Given that miRNAs generally act as inhibitors by promoting the degradation or storage of mRNA transcripts, thereby suppressing protein translation, this suggests that the repression of these miRNAs would effectively function to activate mTORC1 in marsupials during torpor. This is in contrast to the results in ground squirrels showing that *miR-193a*, which directly inhibits translation of mTOR and P70S6K gene transcripts, is upregulated by ~10-fold during torpor, indicating evidence of miRNA mediated inhibition of mTOR transcript translation in hibernating ground squirrels [58,59]. 

Going forward, mTORC1 will continue to be a popular topic of interest in mammalian hibernation. Early studies with ground squirrels, bears, and bats have indicated that suppression of mTORC1 is evident in these seasonal hibernators. However, recent work with species that undergo opportunistic daily torpor such as lemurs and marsupials show that mTORC1 is not necessarily suppressed when these animals are under short-term dormancy. As mentioned earlier, the disparity between the duration, and perhaps the depth of metabolic depression that is experienced between seasonal versus daily torpor may be a key factor in influencing relative mTORC1 activity. In seasonal hibernators, suppression of mTORC1 acts as an important pathway to inhibit active protein biosynthesis, and this may serve as a critical mechanism to turn off ATP expensive cellular processes as a means to conserve energy and promoting survival in a long-term hypometabolic state. 

### 2.2. mTORC1 Regulation in Hypoxia/Anoxia Tolerant Models

Hypoxia is a condition where there is an inadequate supply of oxygen to meet normal demands in order to maintain cellular homeostasis. Oxygen limitation/deprivation has been shown to antagonize mTORC1 activity by activating the TSC1-TSC2 inhibitory complex, through a mechanism that signals through the AMPK protein (Figure 1) [60]. Given that aerobic respiration is central to the metabolic processes of most species on Earth, it is not surprising that mTORC1 activity would be suppressed under a low oxygen cellular environment. Hypoxia-induced mTORC1 inhibition is accompanied by reduced phosphorylation of 4E-BP1 to achieve a state of cap-dependent translational arrest [61]. Whereas continuous oxygen supply is crucial to life for most animals, there are exceptional species that tolerate prolonged periods of hypoxia or even anoxia (complete deprivation of oxygen). The naked mole rat *Heterocephalus glaber* is one example of a hypoxia-tolerant species. These small mammals spend their lives in subterranean tunnels and display unique physiological adaptations to unpredictable changes in their underground habitat [62]. Naked mole rats are eusocial animals that live in large colonies underground, where they experience frequent fluctuations in their oxygen availability and are chronically exposed to a hypoxic environment. Laboratory studies have shown that naked mole rats can survive for several hours at 3% oxygen and up to 18 min in total oxygen deprivation [63,64]. Under hypoxic conditions, the body temperature of these animals is allowed to fall slightly from ~32 °C at 21% oxygen to 29 °C at 3% oxygen, suggesting that these animals can remain metabolically active in part by reducing oxygen use for thermogenesis [63]. In a recent study, the Akt/mTOR pathway was investigated in naked mole rats given 4-hour exposure to 7% oxygen (compared with 21% oxygen controls). Across the 4 tissues examined (brain, liver, muscle, lung), p-mTOR (Ser-2448) was elevated in brain and lung, whereas p-Akt (Ser-473) was elevated in the liver [18]. Consistently, p-4E-BP1 was also elevated in brain and lung, suggesting that mTORC1 is activated in these tissues under hypoxia. Activation of mTORC1 under low oxygen conditions may serve as a protective mechanism in naked mole rats by upregulating metabolic reprogramming to enhance glycolytic capacity. A recent study showed that exposure to anoxia activates the glycolytic pathway in naked mole rats by increasing the anaerobic metabolism fueled by fructose in the brain [64]. This switch to fructose-fueled metabolism is highly unusual and is hypothesized to be a protective mechanism for naked mole rats by providing ATP to essential tissues to maintain survival under hypoxic and/or anoxic environments [64]. One potential mechanism driving the increase in glycolytic flux in the brain may be linked to the increase in mTORC1 activation. A well-characterized function of mTORC1 is to stimulate glycolysis; this signaling pathway requires the activation of the hypoxia-inducible factor-1 alpha (HIF-α) transcription factor that drives the gene expression of several glycolytic enzymes and glucose transporters [65]. In support of this, transcript levels of glucose transporter 5 (a selective fructose transporter) and ketohexokinase (converts fructose into the glycolytic intermediate fructose-1-phosphate) are highly overexpressed in the naked mole rats compared to mice. Not surprisingly, protein levels of HIF-1α are elevated over 3-fold in brain of naked mole rats while under hypoxia [66]. Together, these studies demonstrate a mechanism by which activation of mTORC1 in the brain of naked mole rats initiates metabolic rewiring mediated through HIF-1α to increase anaerobic glycolytic flux as a means to promote energy production under severe hypoxia. Interestingly, activation of mTORC1 under oxygen deprivation was also observed in red-eared slider turtles (*Trachemys scripta elegans*). Red-eared sliders are anoxia tolerant and can survive for several days without breathing oxygen at elevated temperatures (~16–18 °C) or several months at 3 °C during underwater hibernation in the winter [67,68]. One study measuring de novo protein synthesis in red-eared sliders indicated that the rate of radiolabeled ^35^S-methionine incorporation was not substantially reduced after 20 hours of anoxia exposure, and ^35^S-methionine was actually elevated in select tissues including the liver and heart [69]. Biochemical studies have shown that levels of p-Akt (Ser-473) were elevated in the liver and muscle of red-eared slider turtles during anoxia, meanwhile, p-mTOR was maintained at a similar level compared to controls in the liver but increased by ~4.5-fold in the skeletal muscle [17]. Consistent with mTORC1 being activated during oxygen deprivation, levels of p-S6 protein and p-4E-BP1 were also highly increased in turtles after 20 hours of anoxia exposure [17]. 

Together, studies in hypoxia and anoxia-tolerant animals suggest that the mTOR protein complex is activated during periods of oxygen deprivation (Figure 2). This is in contrast with studies in human cells where hypoxia exposure triggers the activation of TSC2 to inhibit mTORC1. Interestingly, in the red-eared sliders, hypoxia exposure does not affect the levels of p-TSC2 (Thr-1462), suggesting that additional regulatory mechanisms may be in place to prevent hypoxia-mediated TSC2 activation in order to maintain mTORC1 function. Studies with naked mole rats indicate that mTORC1 activation may serve to promote anaerobic glycolysis as a means to provide ATP to essential tissues including the heart and brain during periods of oxygen deprivation. A similar increase in glycolytic flux is also observed in turtles, as supported by a recent transcriptomic study showing that gene expression of several glycolytic enzymes was highly up-regulated after 20 hours of anoxia exposure [70]. Consistent with an increase in anaerobic glycolysis, levels of lactate were also highly elevated in tissues of both naked mole rats and turtles during periods of anoxia exposure [64,71]. Overall, studies in these two hypoxia/anoxia tolerant models indicate that mTORC1 is activated during periods of oxygen deprivation and this functions to potentially reprogram metabolic pathways to favor anaerobic metabolism as the means to provide essential energy to vital organs.

### 2.3. mTORC1 Regulation in Animal Models of Estivation

The third group of stress-tolerant animals for which mTORC1 involvement has been extensively characterized are those that undergo estivation, which is a state of prolonged dormancy aimed at enduring arid environmental conditions [72]. *Xenopus laevis* is a model organism commonly used for studying vertebrate developmental biology and embryology, but in its native environment of sub-Saharan Africa, this frog frequently undergoes bouts of estivation to survive seasonal arid conditions [72]. As water dries up in their natural habitat, *X. laevis* are forced to estivate by burrowing into the subsoil of evaporating/drying ponds. In this desiccating environment, a major physiological consequence for the frogs is a loss of up to ~35% of body water while in estivation [73]. A recent study using dehydration to induce estivation in *X. laevis* showed that 16–19% water loss can trigger a ~50% reduction in the levels of phosphorylated IGFR (Tyr-1135/1136) in the brain and heart of *X. laevis*. As mentioned earlier, IGFR is activated by phosphorylation in response to growth factor or insulin stimulation that then triggers downstream activation of the Akt/mTORC1 pathway (Figure 1). Consistent with a reduction in the insulin signaling pathway, levels of p-Akt (Ser-473) are also substantially decreased in brain and heart of *X. laevis,* and this is further supported by a 40% decrease in p-mTOR (Ser-2448) levels [19]. Changes in phosphorylation of these proteins suggest that the insulin signaling axis is suppressed in *X. laevis* under dehydration stress. Dehydration-induced suppression of Akt/mTORC1 activity has previously been reported in mammalian cells where exposure to hyperosmotic conditions inhibited various IGFR downstream functions including Akt activation, glucose uptake, and protein translation via P70S6K and 4E-BP1 phosphorylation [74,75]. It is plausible that *X. laevis* takes advantage of the suppressive effect dehydration has on insulin signaling as a potential mechanism to shut off protein synthesis during estivation and thereby conserve cellular resources to promote long term survival. 

### 2.4. mTORC1 in Genetic Models of Metabolic Arrest 

A unique hypometabolic state displayed by the nematode *Caenorhabditis elegans* is the dauer diapause. During larval development, if *C. elegans* encounter unfavorable conditions such as food deprivation or high temperature, they enter into dauer arrest that serves as an alternative developmental stage [76]. While in dauer, *C. elegans* can survive for months without food and exhibit increased resistance to various external stressors including heat, oxidative stress, and starvation [76]. This form of metabolic adaptation in response to environmental and nutrient stress is comparable to hibernation where the animal enters a state of quiescence until surrounding habitats improve. The biochemical regulation of mTORC1 signaling in *C. elegans* has not been explored, but studies have characterized the genetic requirement for mTORC1 in dauer formation. The *C. elegans* Raptor protein is encoded by a gene called *daf-15*, named for the abnormal DAuer Formation phenotype observed in worms with mutations in this gene [77]. Loss of function to *daf-15* induces a dauer-like larval arrest, a phenotype that is also reproduced when the *C. elegans* mTOR (*let-363/TOR*) gene is knocked-down by RNAi [78]. Loss of function mutation of the *C. elegans* IGFR gene (*daf-2*) is also a strong inducer of dauer formation, suggesting that the insulin/mTORC1 axis is prominently involved in dauer regulation. At 25 °C, nearly 100% of *daf-2* mutants constitutively enter dauer but can develop normally when cultivated at a temperature range of 15–20 °C [79]. At 20 °C, where the penetrance of *daf-2* dauer phenotype is only partial, RNAi knockdown of *let-363/TOR* in *daf-2* mutants has been shown to further enhance dauer entry from 4.6% to 17.9% (percentage of worms displaying the dauer phenotype) [80]. Collectively, studies with *C. elegans* indicate that perturbations to the insulin/mTORC1 signaling axis through depletion of IGFR, Raptor, or mTOR directly leads to the onset of a quiescence state that is comparable to other dormant phenotypes such as hibernation that are also characterized by mTORC1 inhibition. Similar to the mammalian mTOR, *let-363/TOR* is also a regulator of global translational activity in *C. elegans*, although it remains to be determined whether inhibiting protein biosynthesis contributes to or is directly involved in dauer formation [81]. 

## 3. Perspectives

Since its initial discovery in the 1990s, mTOR has emerged as a central protein of interest in thousands of studies across various model organisms and many mammalian cell models. The biochemical and molecular discoveries about mTOR have revealed that this protein complex is intimately involved in regulating organismal homeostasis in the context of determining cellular growth and reproduction, stress survival, aging, and disease progression [12,82]. 

In this review, we highlighted recent advances in understanding how mTORC1 contributes to the establishment of a hypometabolic dormancy, which is an adaptive strategy utilized by multiple animal species when faced with extreme environments that threaten survival. These natural models of metabolic rate depression offer unique insights into how the mTORC1 network is differentially controlled in a stress-dependent manner. For example, initial studies of seasonal hibernators showed that the insulin-Akt-mTORC1 signaling axis is suppressed across multiple tissues in ground squirrels during hibernation, and this acts to shut off protein biosynthesis as a means of conserving metabolic fuel/energy [16,29,34,35]. This is supported by modifications to mTORC1 downstream targets such as the S6 ribosomal proteins and 4E-BP1 towards creating a state of translational arrest [16,35]. Interestingly, recent studies in tropical hibernators such as lemurs and marsupials show that daily torpor bouts are not associated with a general reduction in mTORC1 activity, suggesting that multiple factors such as torpor body temperature, and the depth or duration of torpor, are likely factors that influence mTORC1 activity during metabolic depression [51,57]. Perhaps the more surprising findings are in hypoxia/anoxia tolerant models where mTORC1 has been shown to be activated during periods of oxygen deprivation. In the naked mole rat and red-eared slider turtle, levels of p-Akt, p-mTOR, or mTORC1 downstream targets p-S6 and p-4E-BP1 are increased in various tissues during periods of hypoxia, suggesting that oxygen deprivation may actually trigger mTORC1 activation in these animals. The activation of mTORC1 in hypoxia-tolerant animals may be linked to its direct role in promoting glycolysis via HIF-1α, since a recent study demonstrates that an increase in fructose-driven anaerobic glycolysis is an essential source of ATP for the heart and brain of naked mole rats during oxygen deprivation [64]. 

Going forward, an important area for continuing mTOR research includes studying the function of the less well characterized mTORC2 to determine its role in regulating stress adaptation. For example, recent work with cultured cells has demonstrated that mTORC2 is activated by AMPK during acute energetic stress as a protective mechanism to inhibit apoptosis and promote cell survival [83]. This would be an intriguing area of study in hibernators that show activation of anti-apoptotic signals during torpor [84,85]. 

In conclusion, biochemical and molecular studies of metabolically flexible and stress-tolerant animals present an unparalleled opportunity to advance our understanding of how mTOR function is controlled under extreme cellular conditions that are not easily replicated in cultured cells. Overall, we expect mTOR signaling will continue to be a popular and important topic of study in other yet-to-be-characterized animal models of metabolic plasticity. New insights from novel metabolic situations will continue to add to our knowledge of the functions of mTOR in regulating cellular growth, development, and survival during adaption to environmental stress.

## Data Availability

Not applicable.
